# Activation of Aldehyde Dehydrogenase 2 Ameliorates Glucolipotoxicity of Pancreatic Beta Cells

**DOI:** 10.3390/biom11101474

**Published:** 2021-10-06

**Authors:** Shiau-Mei Chen, Siow-Wey Hee, Shih-Yun Chou, Meng-Wei Liu, Che-Hong Chen, Daria Mochly-Rosen, Tien-Jyun Chang, Lee-Ming Chuang

**Affiliations:** 1 Department of Internal Medicine, National Taiwan University Hospital, Taipei 100, Taiwan; joe8214@gmail.com (S.-M.C.); d91448003@ntu.edu.tw (S.-W.H.); keepup1209@gmail.com (S.-Y.C.); bioterry1028@gmail.com (M.-W.L.); leeming@ntu.edu.tw (L.-M.C.); 2 Department of Chemical and Systems Biology, Stanford University School of Medicine, Stanford, CA 94305, USA; chehong@stanford.edu (C.-H.C.); mochly@stanford.edu (D.M.-R.); 3 School of Medicine, College of Medicine, National Taiwan University, Taipei 100, Taiwan

**Keywords:** aldehyde dehydrogenase 2 (ALDH2), glucolipotoxicity, beta cell function, Alda-1

## Abstract

Chronic hyperglycemia and hyperlipidemia hamper beta cell function, leading to glucolipotoxicity. Mitochondrial aldehyde dehydrogenase 2 (ALDH2) detoxifies reactive aldehydes, such as methylglyoxal (MG) and 4-hydroxynonenal (4-HNE), derived from glucose and lipids, respectively. We aimed to investigate whether ALDH2 activators ameliorated beta cell dysfunction and apoptosis induced by glucolipotoxicity, and its potential mechanisms of action. Glucose-stimulated insulin secretion (GSIS) in MIN6 cells and insulin secretion from isolated islets in perifusion experiments were measured. The intracellular ATP concentrations and oxygen consumption rates of MIN6 cells were assessed. Furthermore, the cell viability, apoptosis, and mitochondrial and intracellular reactive oxygen species (ROS) levels were determined. Additionally, the pro-apoptotic, apoptotic, and anti-apoptotic signaling pathways were investigated. We found that Alda-1 enhanced GSIS by improving the mitochondrial function of pancreatic beta cells. Alda-1 rescued MIN6 cells from MG- and 4-HNE-induced beta cell death, apoptosis, mitochondrial dysfunction, and ROS production. However, the above effects of Alda-1 were abolished in *Aldh2* knockdown MIN6 cells. In conclusion, we reported that the activator of ALDH2 not only enhanced GSIS, but also ameliorated the glucolipotoxicity of beta cells by reducing both the mitochondrial and intracellular ROS levels, thereby improving mitochondrial function, restoring beta cell function, and protecting beta cells from apoptosis and death.

## 1. Introduction

Insulin resistance and progressive pancreatic beta cell dysfunction are the main features of type 2 diabetes mellitus, most likely owing to a vicious cycle involving the accumulation of toxic aldehydes and relative oxidative stress [1]. It is well known that chronic hyperglycemia and hyperlipidemia, also known as glucolipotoxicity, are harmful to beta cell function. Under glucolipotoxicity, the fine balance between the levels of pro-oxidants and antioxidants in pancreatic beta cells is disturbed, which leads to a chronic oxidative stress that subsequently contributes to impaired glucose-stimulated insulin secretion (GSIS) [2,3]. Pancreatic beta cells are particularly susceptible to oxidative stress, which contributes to beta cell dysfunction and cell death [4]. The decreases in antioxidant defense, hyperglycemia, inflammation, and obesity contribute to the accumulation of toxic aldehydes, including glyoxal, methylglyoxal (MG), glycolaldehyde, and 4-hydroxynonenal (4-HNE), among others [5]. The elevated levels of toxic aldehydes can damage carbohydrates, amino acids, and lipids, leading to the formation of reactive carbonyl compounds (RCCs), which further react with macromolecules and yield advanced glycation end products (AGE) or advanced lipoxidation end products (ALE). AGEs bind to the cell surface receptor for the advanced glycation end products (RAGE) and induce the phosphorylation of PKC and subsequent activation of NADPH oxidase, thereby leading to theexcessive intracellular reactive oxygen species (ROS) formation [5] and the activation of key transcriptional factors, such as NFκB, AP1, Nrf2, HSF1, PDX1 and FOXO1 [6]. The alteration of transcriptional factors by toxic aldehydes modifies the gene expression of proinflammatory cytokines, detoxifying genes, heat shock proteins (HSP), insulin gene expression, and beta cell proliferation [6].

Cohen et al. studied the effects of 4-HNE on the rat islets of Langerhans and the rat insulinoma INS-1E beta cell line and demonstrated that exogenously added, dose-dependent 4-HNE induced apoptosis and cell death [7]. MG is an intracellularly formed α-ketoaldehyde that is involved in the formation of AGEs. The accumulation of MG contributes to glucotoxicity and mediates beta cell apoptosis [8].

Aldehyde dehydrogenase 2 (ALDH2) is located in the mitochondria, where it plays a major role in acetaldehyde detoxification in humans and the detoxification of ROS-generated aldehyde adducts [9]. ALDH2 transgenic mice showed an enhanced acetaldehyde detoxification following chronic alcohol intake, leading to an improved whole-body glucose tolerance, cardiac glucose uptake and insulin signaling at the receptor and post-receptor levels [10]. The ALDH2 activator, Alda-1, significantly accelerated adipocyte differentiation in 3T3-L1 cells through the regulation of PPARγ transcriptional activity [11]. Furthermore, the administration of Alda-1 decreased 4-HNE concentration [12] and attenuated cardiac [12,13] and renal damage [14] induced by ischemia-reperfusion injury through increased oxidative stress. Therefore, it is reasonable to hypothesize that the administration of an ALDH2 activator can ameliorate beta cell dysfunction and apoptosis induced by glucolipotoxicity. In this study, we showed that the prototype ALDH2 activator (Alda-1) significantly enhanced insulin secretion by improving the mitochondrial function of the beta cells. Moreover, Alda-1 ameliorated either MG- or 4-HNE-induced beta cell apoptosis by alleviating the production of superoxide from the mitochondria and the cytoplasm, subsequently leading to improved mitochondrial function, decreasing beta cell apoptosis and death.

## 2. Materials and Methods

### 2.1. Chemicals

Alda-1 was kindly provided by Dr. Wen-Jin Yang (Foresee Pharmaceuticals Co., Ltd., Taipei, Taiwan). Appropriate stock concentrations of the compounds tested in this study were prepared in 100% dimethyl sulfoxide (DMSO). The 4-HNE was purchased from Merck Millipore (Temecula, CA, USA). MG and other chemicals were purchased from Sigma-Aldrich (St. Louis, MO, USA). Bovine serum albumin-palmitate saturated fatty acid complex (PA) and bovine serum albumin (BSA) control were purchased from Cayman Chemical Company (Ann Arbor, MI, USA).

### 2.2. Cell Culture

MIN6 cells, kindly provided by Prof. Susumu Seino (Kobe University, Kobe, Japan), were used between passages 40 and 50 and grown in Dulbecco’s modified Eagle’s medium (DMEM) containing 10% (vol/vol) fetal bovine serum at 37 °C with 5% CO_2_. Mouse *Aldh2* short-hairpin RNA and scramble control lentiviral clones were purchased from the National RNAi Core Facility at Academia Sinica (Taipei, Taiwan). MIN6 cells were infected with the viral supernatants according to the manufacturer’s protocol and further selected with 2 μg/mL puromycin.

### 2.3. Measurement of GSIS

The cells were seeded and incubated for 24 h prior to the experiment. Next, the previous medium was removed and replaced with Krebs Ringers buffer (KRB) containing 3.3 mM glucose, and the cells were incubated at 37 °C for 2 h. Then, this medium was replaced with KRB, containing either 3.3 or 16.7 mM glucose for 30 min. Then, the medium was collected, and insulin concentration was measured using Ultrasensitive Rat Insulin ELISA (Mercodia Developing Diagnostics, Uppsala, Sweden).

### 2.4. Islet Isolation and Perifusion Study

Pancreatic islet cells were isolated from 16-week-old mice by collagenase digestion, as described previously [15]. Islet cells were cultured at 37 °C, 5% CO_2_, pH 7.4, in RPMI 1640 containing 10% fetal bovine serum, 10 mM glucose, 180 μM penicillin, and 68.6 μM streptomycin, and the islets were used for experiments 2–3 days after isolation.

The GSIS of the isolated islets was measured in perifusion experiments. In brief, KRB buffer containing 2 mM glucose was pumped at a rate of 0.5 mL/min around islets loaded into 300 μL plastic chambers. A total of 100 isolated islets were perifused for 10 min equilibration period (2 mM glucose containing KRB perifusate as a basal concentration), which was followed by a 40 min period of perifusion with high glucose (16.7 mM glucose containing KRB perifusate). Fractions were collected every 2 min using an automatic fraction collector. Insulin concentration in the perifusate was measured using Ultrasensitive Rat Insulin ELISA (Mercodia Developing Diagnostics, Uppsala, Sweden). Quantitation of area under the curve (AUC) for first-phase (10–20 min) and second-phase (20–50 min) insulin release was performed as described previously [16].

### 2.5. Intracellular ATP Assay

The cells were seeded and incubated for approximately 24 h prior to the experiment. Next, the previous medium was removed and replaced with KRB containing 3.3 mM glucose, and the cells were incubated at 37 °C for 2 h. This medium was then replaced with KRB, containing either 3.3 or 16.7 mM glucose, with or without Alda-1 at different concentrations as indicated for 30 min. The ViaLight^®^ Plus Kit (Lonza, Rockland, MA, USA) was used to examine the level of ATP (adenosine5’-triphosphate) in cells following the manufacturer’s instructions. In brief, 100 μL of assay buffer was added to each well of a 96-well plate containing 50 μL of lysis buffer. After incubating the plate for 2 min at room temperature, luminescence was measured using a Spark^®^ multimode microplate reader (TECAN, Männedorf, Switzerland).

### 2.6. Cellular Oxygen Consumption Rate (OCR)

Cellular respiration was measured using a Seahorse XF24 analyzer (Seahorse Biosciences, North Billerica, MA, USA). MIN6 cells were seeded in Seahorse XF 24-well culture plates at 5 × 10^4^ cells/well in DMEM growth medium and allowed to adhere overnight. Before the experiment, the medium was replaced with 750 μL of an unbuffered medium, and the cells were equilibrated for 1 h at 37 °C in a CO_2_-free incubator. The OCR was determined for 130 min.

### 2.7. Cell Viability Assay

The cells were seeded and incubated for 48 h prior to the experiment. The next day seeded cells were pre-treated with Alda-1 for 1 h and then incubated in medium containing MG or 4-HNE under a 5% CO_2_/air atmosphere in an incubator at 37 °C. For co-treatment of high glucose and fatty acid, seeded cells were pre-treated with Alda-1 for 1 h and then incubated in a medium containing the indicated concentration of glucose or PA for 72 h. Following treatment, cell viability was determined using the Alamar Blue assay (BIO-RAD, Hercules, CA, USA).

### 2.8. Apoptosis Detection with 7-AAD

Apoptosis was determined using the FITC Annexin V Apoptosis Detection Kit with 7-AAD (BioLegend, #640922) according to the manufacturer’s instructions. After the incubation of MG or 4-HNE for 24 h, cells were trypsinized, transferred to 1.5 mL tubes, and washed twice with phosphate-buffered saline (PBS). Then, the cells were resuspended in Annexin V binding buffer and transferred to flow cytometry tubes. FITC Annexin V and 7-AAD were added to the cells for 30 min and measurements were recorded using a FACSVerse flow cytometer (BD Biosciences, Franklin Lakes, NJ, USA). Data were analyzed using the Flow Jo 7.6 software (BD Biosciences, Franklin Lakes, NJ, USA).

### 2.9. Detection of Mitochondrial and Intracellular ROS

For superoxide detection, MitoSOX Red (Thermo Fisher Scientific, M36008, Waltham, MA, USA) and CellROX Green (Thermo Fisher Scientific, C10444, Waltham, MA, USA) were used to label the mitochondrial and intracellular ROS. Following treatment, ROS detection was performed according to the manufacturer’s protocol, and fluorescence was detected using a FACSVerse flow cytometer.

### 2.10. Western Blotting

Cells were lysed in radioimmunoprecipitation assay (RIPA) buffer (Merck Darmstadt, Germany) containing protease and phosphatase inhibitors (Roche Applied Science, Germany). The cell lysates were centrifuged at 12,000× *g* for 10 min at 4 °C to remove insoluble materials. The samples were subjected to sodium dodecyl sulfate-polyacrylamide gel electrophoresis, transferred to a polyvinylidene fluoride (PVDF) membrane and probed with the indicated antibodies, including anti-poly (ADP-ribose) polymerase (PARP), anti-BCL-2, anti-MCL1, anti-p53, anti-Caspase-8, anti-ALDH2, anti-β-actin (GeneTex, GTX100573, GTX100064, GTX102026, GTX102965, GTX110723, GTX101429, and GTX109639, Irvine, CA, USA), anti-phospho-Akt/Akt, anti-cleavedCaspase-3/Caspase-3 (Cell Signaling Technology, CST4060S/9272S, CST9664S/9665S, Danvers, MA, USA), and anti-Bax (Merck Millipore, 04-434, Burlington, MA, USA). Proteins were visualized using enhanced chemiluminescence (Merck Millipore, Burlington, MA, USA). 

### 2.11. Statistical Analysis

Data were presented as mean ± standard error of the mean (SEM). Multiple groups were compared using the Kruskal–Wallis test and post hoc analysis was conducted using Dunn’s multiple comparisons test. For comparison between two groups, the Mann–Whitney U test was used. For all data, statistical significance was set at *p* < 0.05. GraphPad Prism (version 7.0) was used for all statistical analyses.

## 3. Results

### 3.1. ALDH2 Activator Enhanced GSIS

**Figure 1:**
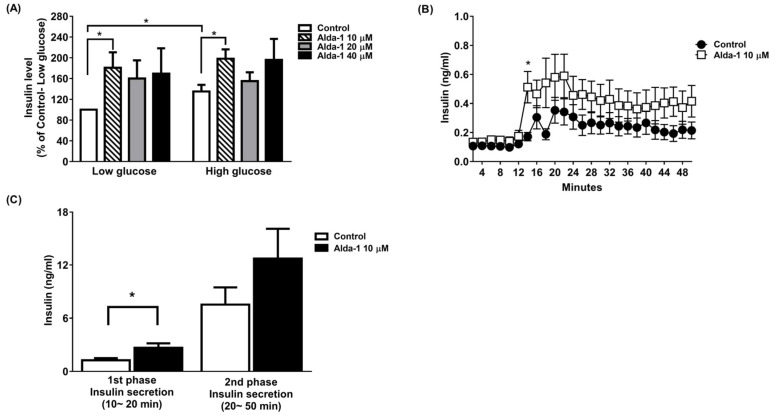
Alda-1 promotes insulin secretion in MIN6 pancreatic beta cells. (**A**) Insulin secretory response in MIN6 cells exposed to different doses of Alda-1 under low (3.3 mM) and high glucose (16.7 mM) concentrations (*n* = 3/group). (**B**) Glucose-stimulated insulin secretion in ex vivo perifused islets in the absence and presence of 10 μM Alda-1. (**C**) First and second phase insulin secretions were measured from the islet perifusion study (details in the Method section). (*n* = 14 in the control group, *n* = 15 in the Alda-1 10 μM group). Data are presented as mean ± SEM * *p* < 0.05 versus the control group.

We tested different doses (10, 20, and 40 μM) of ALDH2 activator Alda-1 on the enhancement of GSIS, and found that Alda-1 at 10 μM potentiated static insulin secretion under both low (3.3 mM) and high (16.7 mM) glucose concentrations in cultured MIN6 cells (Figure 1A).

To further study GSIS in primary islets, an ex vivo primary islet perifusion study was performed. Alda-1 at 10 μM significantly promoted GSIS at 4 min (i.e., 14 min during the perifusion study) after switching the perifusates from a basal to a high glucose concentration (16.7 mM), and continuously increased insulin secretion during the course of the islet perifusion study (Figure 1B). The first phase (0–10 min during high glucose perifusion) and second phase (10–40 min during high glucose perifusion) of insulin secretion were analyzed, and Alda-1 significantly potentiated first-phase insulin secretion (Figure 1C).

### 3.2. ALDH2 Activator Improved Mitochondrial Function of Pancreatic Beta Cells

**Figure 2:**
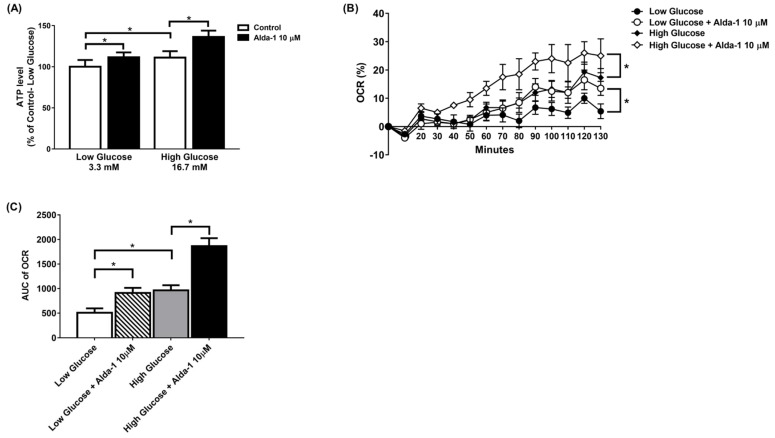
Alda-1 improves mitochondrial function. (**A**) ATP level in response to 10 μM Alda-1 in MIN6 cells under low (3.3 mM) and high glucose (16.7 mM) concentrations. (**B**) Effect of Alda-1 on oxygen consumption rate (OCR) in MIN6 beta cells under low (3.3 mM) and high glucose (16.7 mM) concentrations. (**C**) The area under curves (AUC) of the OCR. Data are presented as mean ± SEM of three independent experiments (*n* = 3 per group). * *p* < 0.05 versus the control group with *p*-for-trend analysis and Student’s *t*-test, respectively.

The ALDH2 activator Alda-1 significantly increased the intracellular ATP content in MIN6 cells cultured under both low and high glucose conditions. (Figure 2A). To further explore the changes in the mitochondrial function of the MIN6 cells under different culture conditions, we employed a Seahorse XF analyzer to measure the OCR. The data showed that Alda-1 increased the OCR for both low and high glucose incubation conditions, which was significant for p-for-trend analyses (Figure 2B). We also compared the AUC of the OCR for MIN6 cells under different conditions. As shown in Figure 2C, Alda-1 treatment significantly increased the AUC of OCR in MIN6 cells under both low and high glucose conditions (Figure 2C). Notably, the effect of Alda-1 on mitochondrial function was independent of ambient glucose concentrations (Figure 2B,C).

### 3.3. Alda-1 Rescued MIN6 Cells from MG- and 4-HNE- Induced Beta Cell Death and Apoptosis

**Figure 3:**
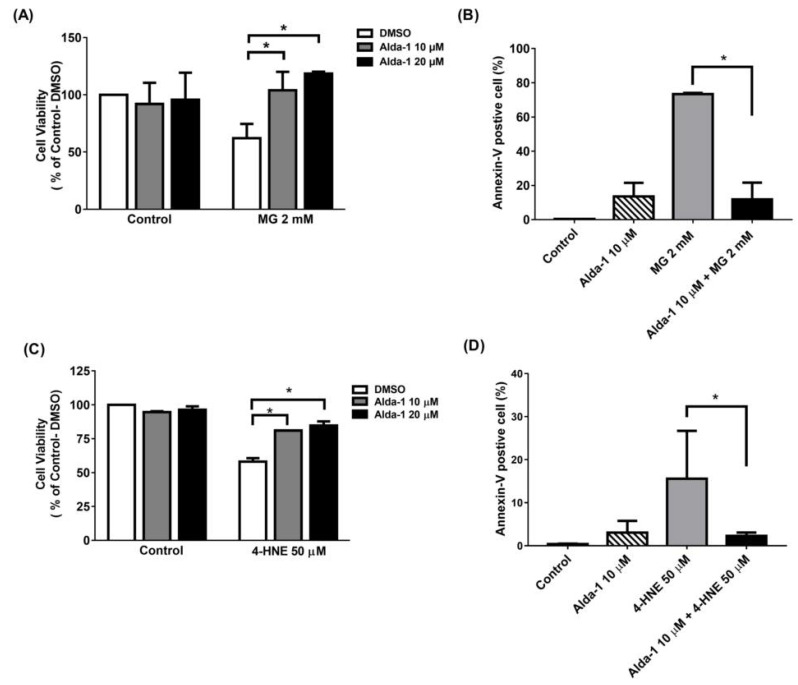
Alda-1 rescues the cell death induced by glucotoxicity and lipotoxicity via anti-apoptotic effect. (**A**) Cell viability in the control and Alda-1-treated MIN6 cells in the absence and presence of 2 mM methylglyoxal (MG). (**B**) The percentages of Annexin-V-positive and AAD-7-negative MIN6 cells treated with or without Alda-1 in the absence and presence of 2 mM methylglyoxal (MG) for 24 h. (**C**) Cell viability in control and Alda-1-treated MIN6 cells in the absence and presence of 50 μM 4-Hydroxynonenal (4-HNE). (**D**) The percentages of the Annexin-V-positive and AAD-7-negative MIN6 cells treated with or without Alda-1 in the absence and presence of 50 μM 4-HNE for 24 h. Data are presented as mean ± SEM of three independent experiments (*n* = 3 per group). * *p* < 0.05.

To investigate the effect of the ALDH2 activator on the improvement of beta cell survival, we measured MIN6 cell survival upon MG and 4-HNE exposure, mimicking glucolipotoxicity conditions. We found that Alda-1 dose-dependently rescued cell survival when MIN6 cells were treated with 2 mM MG (Figure 3A) or 50 μM 4-HNE (Figure 3C). To understand the mechanism underlying cell survival, we used flow cytometry with Annexin V to study the potential effect of Alda-1 on cell apoptosis and/or necrosis. As shown in Figure 3B,D, the pretreatment of MIN6 cells with Alda-1 significantly decreased MG- and 4-HNE-induced beta cell apoptosis.

### 3.4. Alda-1 Rescued MG- and 4-HNE-Induced Mitochondrial Dysfunction in Beta Cells

**Figure 4:**
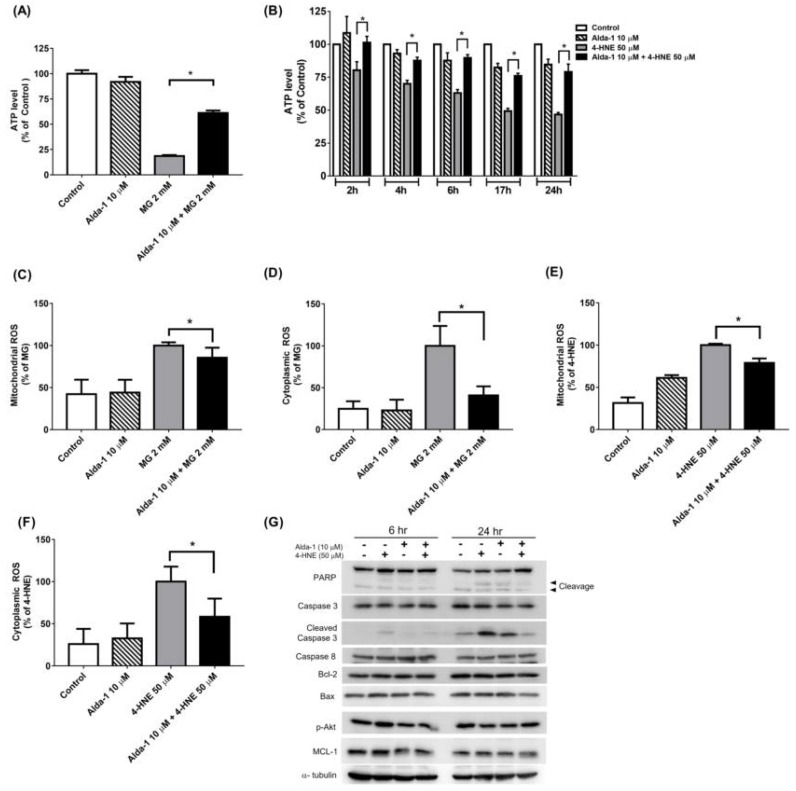
Alda-1 ameliorates mitochondrial dysfunction induced by glucotoxicity and lipotoxicity. The ATP levels in the control and Alda-1-treated MIN6 cells in the absence and presence of 2 mM methylglyoxal (MG) for 24 h (**A**) or 4-HNE for different time periods (**B**). Relative mitochondrial (**C,E**) and cytoplasmic (**D**,**F**) ROS levels normalized to MG or 4-HNE in the control and Alda-1-treated MIN6 cells in the absence and presence of 2 mM MG (**C**,**D**) or 4-HNE (**E**,**F**) for 24 h. (**G**) The signaling pathway of Alda-1 rescuing 4-HNE-induced cell apoptosis. Treatment of Alda-1 improved the beneficial effects on apoptotic protein levels. Data are presented as mean ± SEM of three independent experiments (*n* = 3 per group). * *p* < 0.05.

To further explore the possible mechanisms by which ALDH2 ameliorates MG- and 4-HNE-induced beta cell damage, we assessed the mitochondrial function by measuring the intracellular ATP concentration. MG significantly decreased the intracellular ATP concentration, and the pretreatment with Alda-1 significantly reversed this effect (Figure 4A). Consistently, 4-HNE significantly decreased intracellular ATP concentration in a time-dependent manner, and pretreatment with Alda-1 also significantly restored the intracellular ATP concentration in a time-dependent manner (Figure 4B).

### 3.5. Alda-1 Ameliorated MG- and 4-HNE-Increased Oxidative Stress in Mitochondria and Cytoplasm of Beta Cells

To explore how Alda-1 improves the oxidative stress in MIN6 cells exposed to glucolipotoxicity, we measured the levels of the mitochondrial and cytoplasmic ROS by determining the MitoSox and CellRox fluorescence intensity via flow cytometry. Alda-1 treatment ameliorated MG-induced ROS generation in both the mitochondrial (Figure 4C) and cytoplasmic (Figure 4D) fractions. The similar effects of Alda-1 were also observed in the 4-HNE-treated group (Figure 4E,F). These data showed that Alda-1 administration ameliorated mitochondrial dysfunction by alleviating the oxidative stress induced by glucotoxicity and lipotoxicity. To evaluate the effect of Alda-1 on apoptosis, we measured the protein expression of PARP, Caspase-3, cleaved Caspase 3, Bcl-2, Bax, Caspase 8, p-Akt, and MCL-1 by Western blotting. The expression of cleaved Caspase 3, PARP and Bax was significantly increased after 4-HNE damage, whereas treatment with Alda-1 dramatically mitigated the expression levels (Figure 4G). Treatment with Alda-1 did not affect the anti-apoptotic proteins (MCL-1 and Bcl-2). The results showed that the treatment with Alda-1 improved the beneficial effects on apoptotic protein levels.

### 3.6. Potentiation Effect on Insulin Secretion and Mitochondrial Function of ALDH2 Activator Was Abolished in Aldh2 Knockdown MIN6 Cells

**Figure 5:**
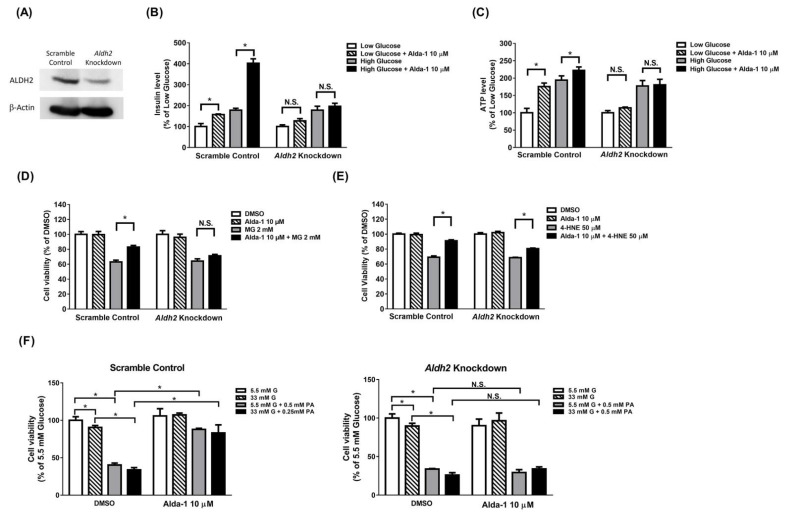
Aldh2 knockdown abolished the anti-apoptotic effects of ALDH2 activator in MIN6 cells. (**A**) The knockdown efficiency of shRNA on ALDH2 expression was analyzed by Western blot. Effect of Alda-1 on scramble control and *Aldh2* knockdown MIN6 cells on (**B**) Insulin secretion and (**C**) ATP levels in low (3.3 mM) and high glucose (16.7 mM) concentrations. Effect of Alda-1 on cell viability in the presence of (**D**) 2 mM methylglyoxal (MG) or (**E**) 50 μM 4-HNE. Data are presented as mean ± SEM (*n* = 3 per group). (**F**) Incubation in either high glucose or palmitate alone or with both combinations, to evaluate the direct toxic effects of glucolipotoxicity and the protective effect of Alda-1. The data were normalized to 5.5 mM glucose as the control and represented as percentage of viability. (*n* = 4–6 per group). G: glucose, PA: palmitate. *: *p* < 0.05. N.S.: not significant.

The expression level of Aldh2 was remarkably decreased in the *Aldh2*-knockdown MIN6 cells (Figure 5A). Alda-1 potentiated insulin secretion in the scramble control MIN6 cells cultured under both low and high glucose concentrations. However, the potentiation effects of insulin secretion by Alda-1 were abolished in *Aldh2*-knockdown MIN6 cells (Figure 5B). Similarly, Alda-1 increased the intracellular ATP concentration in MIN6 cells cultured in both low and high glucose concentrations. However, the incremental effect of intracellular ATP levels by Alda-1 treatment was abolished in *Aldh2*-knockdown MIN6 cells (Figure 5C).

### 3.7. Beta Cell Death Evoked by MG and 4-HNE Cannot Be Fully Prevented by ALDH2 Activator in Aldh2 Knockdown MIN6 Cells

Alda-1 prevented MG and 4-HNE from evoking beta cell death in scramble control MIN6 cells. In contrast, the anti-cell death effect of Alda-1 was completely abrogated in the *Aldh2*-knockdown MIN6 cells treated with MG (Figure 5D). Although Alda-1 partially rescued the beta cell viability in *Aldh2*-knockdown MIN6 cells treated with 4-HNE, the percentage of anti-death effect of Alda-1 was much less than that in scramble control MIN6 cells (Figure 5E).

### 3.8. Beta Cell Death Induced by Either Hyperglycemia or Palmitate or Both Cannot Be Fully Prevented by ALDH2 Activator in Aldh2-Knockdown MIN6 Cells

To directly investigate the effect of Alda-1 on rescuing glucotoxicity and lipotoxicity in beta cells, scramble control or *Aldh2*-knockdown MIN6 cells were incubated in normal glucose (5.5 mM), high glucose (33 mM), normal glucose (5.5 mM) with palmitate (0.5 mM), or high glucose (33 mM) with palmitate (0.5 mM) in the presence or absence of Alda-1 (10 μM) for 72 hrs. Either high glucose or palmitate, alone or in combinations, significantly decreased cell viability in both scramble control and *Aldh2*-knockdown MIN6 cells. However, Alda-1 preferentially rescued the cell viability suppressed by palmitate alone or both high glucose and palmitate conditions in scramble control, but not in *Aldh2*-knockdown MIN6 cells (Figure 5F).

## 4. Discussion

In this study, we found that Alda-1, an ALDH2 activator, potentiated insulin secretion in both beta cells and mouse primary islets by improving mitochondrial function. On the other hand, Alda-1 also ameliorated the harmful effects of glucolipotoxicity on beta cells by improving mitochondrial function and reducing ROS production, as well as the apoptosis of beta cells. To the best of our knowledge, this was the first study to demonstrate the rescuing glucolipotoxicity effects of the ALDH2 activator in pancreatic beta cells.

The prevalence of type 2 diabetes mellitus has increased worldwide [17]. Insulin resistance and progressive pancreatic beta cell failure are the main features of type 2 diabetes mellitus [1]. The progressive loss of beta cell mass and the progressive decline in beta cell function are the main pathogeneses leading to the progression of type 2 diabetes [18]. The targets for the treatment of diabetes mellitus remain unsatisfactory despite several classes of anti-diabetic agents used in the clinical setting. Most types of therapy eventually fail as type 2 diabetes is a progressive disorder. Therefore, there is still an unmet medical need for the sustained and effective treatment of type 2 diabetes. High glucose concentration increases the cytosolic ATP level, which induces the closure of K_ATP_ channels and results in cell membrane depolarization, followed by the opening of the voltage-dependent calcium channel. Subsequently, this leads to a Ca^2+^ influx in the cells with increased [Ca^2+]_i_^, thereby promoting the exocytosis of insulin-containing granules [19]. The triggering pathway is essential for the first phase of insulin secretion [19]. In the process of GSIS, the glycolytic flux is tightly coupled to increased mitochondrial oxidative activity, leading to the increased production of ROS [20]. ALDH2 is a nuclear-coded aldehyde oxidase that is localized in the mitochondrial matrix. Many studies confirmed that ALDH2 can decompose the acetaldehyde metabolite 4-HNE and mitigate oxidative damage to the cells induced by acetaldehyde and its metabolites [21]. A recent study reported that fibroblasts of a patient with Alzheimer’s disease (AD) had approximately 25% ALDH2 activity relative to the fibroblasts of a healthy subject. The AD-derived fibroblasts increased mitochondrial ROS production, reduced ATP levels, reduced mitochondrial respiration (OXPHOS), and caused a shift towards glycolysis (ECAR) relative to the fibroblasts derived from healthy subjects. All of the above defects observed in AD-derived fibroblasts were significantly corrected with Alda-1 treatment [22]. Another study also showed that the impairment of ALDH2 accelerated the acquisition of a premature senescent phenotype in endothelial cells, a change likely to be associated with the observed reduction in mitochondrial respiration and its reserved capacity [16]. In this study, the ALDH2 activator Alda-1 potentiated insulin secretion in MIN6 cells and first-phase insulin secretion in primary islets by improving mitochondrial function as indicated by an increase in the intracellular ATP concentration and oxygen consumption rate. The improved mitochondrial function may be caused by the elimination of ROS and subsequent enhancement of insulin secretion via the increased intracellular ATP/ADP ratio. The potentiation effect of Alda-1 on insulin secretion and the intracellular ATP concentrations were abolished in *Aldh2*-knockdown MIN6 cells, indicating that the effect of Alda-1 was mediated through the activation of ALDH2. More interestingly, the intracellular ATP concentrations under conditions of high glucose concentrations were similar to those in *Aldh2*-knockdown MIN6 cells cultured under high glucose concentrations together with Alda-1, suggested that the effect of improving mitochondrial function was caused by the activation of ALDH2 independent of glucose concentration.

The long-term exposure to high concentrations of glucose and non-esterified free fatty acid (NEFA) in beta cells altered membrane fluidity, protein palmitoylation, and ceramide production, which resulted in mitochondrial dysfunction, endoplasmic reticulum (ER) stress, autophagy, and apoptosis [23,24,25,26]. Arachidonic acid and linoleic acid were subjected to peroxidation, resulting in the generation of 4-HNE, which induced apoptosis and cell death in terms of lipotoxicity [27]. ALDH could oxidize 4-HNE to 4-hydroxy-2-nonenoic acid (HNA), which was one of the three major detoxification pathways for converting 4-HNE to a less reactive chemical species [28]. ALDH2, a member of the ALDH family, was exclusively located in the mitochondria [28]. The prototype of the ALDH2 activator, Alda-1, activated the wild type enzyme and restored the activity of the ALDH2*2 mutant enzyme by acting as a structural chaperone [29]. In a recent study, the activation of ALDH2 prevented the cardiac-arrest-induced death of cardiomyocytes from 4-HNE-induced mitochondrial ROS production and the subsequent mitochondrial damage and cell apoptosis [30]. In this study, we demonstrated that Alda-1 ameliorated 4-HNE-induced beta cell death, apoptosis, and mitochondrial, as well as cytoplasmic ROS levels. Moreover, Alda-1 significantly restored the 4-HNE-induced reduction in intracellular ATP concentration in a time-dependent manner. Finally, we also showed that the pretreatment with Alda-1 decreased the expression of apoptotic molecules, such as cleaved PARP, cleaved caspase 3, and Bax. However, the expression of anti-apoptotic molecules, such as MCL-1, Bcl-2, and p-Akt, was not affected by Alda-1. Consistently, the alleviating effect of Alda-1 on beta cell death was abrogated in the *Aldh2*-knockdown MIN6 cells, which validated the effect of Alda-1 on beta cell viability through the activation of ALDH2.

Chronic hyperglycemia leads to the formation of AGE by promoting the non-enzymatic glycation of endogenous proteins, lipids and nucleic acids [31]. MG is an intracellularly formed α-ketoaldehyde, which is an essential source of intracellular AGEs. It is reported that MG suppresses the oxygen consumption rate and decreases intracellular ATP levels in RINm5F beta cells [32]. Several reports also demonstrated that MG or glyoxal reduces the mitochondrial membrane potential, suppresses the activities of respiratory chain complexes, decreases the ATP production, and elevates the ROS levels in different cells [33,34,35]. Moreover, MG increases the intracellular ROS production and lactate levels and decreases the mitochondrial membrane potential and intracellular ATP levels in SH-SY5Y neuroblastoma cells. The MG-induced depletion of ATP and mitochondrial dysfunction can be prevented by the pretreatment with the carbonyl scavengers aminoguanidine and tenilsetam [34]. Although MG is a substrate of ALDH2 [9], the alleviation of MG-induced beta cell death and apoptosis by ALDH2 activation is not reported. In this study, we showed that Alda-1 ameliorated MG-induced beta cell death, apoptosis (Figure 3B), and mitochondrial, as well as cytoplasmic, ROS production. Moreover, Alda-1 restored the MG-suppressed intracellular ATP concentration, suggesting that the activation of ALDH2 improved mitochondrial function. Furthermore, we demonstrated that the rescue effect of Alda-1 on beta cell viability was abolished in *Aldh2* knockdown MIN6 cells, indicating that the effect of Alda-1 on beta cell survival was mediated by the activation of ALDH2.

The high circulating levels of both glucose and free fatty acids are known to induce oxidative stress in beta cells [36,37]. Beta cells are particularly sensitive to oxidative stress due to the low expression levels of antioxidant enzymes [4]. In this study, we demonstrated that Alda-1 not only alleviated MG- and 4-HNE-induced beta cell dysfunction, apoptosis and death, but also ameliorated either palmitate per se or both high glucose- and palmitate-evoked toxic effects. Therefore, the activation of ALDH2 attenuated glucolipotoxicity induced from high glucose and fatty acids, alone and by their toxic byproducts, such as MG and 4-HNE.

Because of the limitation of human islets resources, we only used a beta cell line and primary islets from mice to demonstrate the protective effect of Alda-1 from glucolipotoxicity on beta cells in this study. We will conduct an experiment on human primary islets in the future study. Another limitation of this study was that the MIN6 cells used in the experiments underwent a long-term passage. According to previous observations [38,39], high-passage MIN6 cells lose their ability to secrete insulin in response to glucose, especially with no first-phase insulin secretion and an impaired second-phase GSIS. The phenotypes observed in the high passage MIN6 cells are then similar to patients with an early onset of type 2 diabetes. In our study, we used high-passage MIN6 cells and found a small but significant response of insulin secretion to high glucose (Figure 1A. insulin level in high glucose: 135% of Control-Low glucose, *p* = 0.042). This finding was compatible with previous reports [38,39]. In this circumstance, the ALDH2 activator Alda-1 still potentiated the insulin secretion of MIN6 cells in both low and high glucose concentrations, suggesting a potential for the treatment of type 2 diabetes subjects.

Pancreatic beta cell failure is pivotal to diabetes development [40,41,42] and the preservation of functional beta cells can change the clinical outcome of diabetes [43,44]. However, none of the current anti-diabetic drugs reversed the progression of beta cell dysfunction and death. In this study, we developed a new strategy of preserving beta cell function and cell viability by activating ALDH2 to detoxify glucolipotoxicity-induced ROS production, decreasing mitochondrial function and subsequent beta cell dysfunction, cell apoptosis and death.

## 5. Conclusions

In this study, we revealed that the activator of ALDH2 not only enhanced insulin secretion, but also ameliorated the harmful effects of glucolipotoxicity on beta cells by reducing both the mitochondrial and cytoplasmic ROS levels, improving mitochondrial function, subsequently restoring beta cell function and preventing beta cells from apoptosis and cell death. These data pave the way for the development of novel antidiabetic agents by improving beta cell function and survival to tackle diseases such as type 2 diabetes with progressive beta cell loss.

## Data Availability

The data presented in this study are available on request from the corresponding author.
